# Computational fluid dynamics-based prediction of aortic aneurysm rupture in a patient with chronic aortic dissection

**DOI:** 10.1186/s44215-023-00091-w

**Published:** 2023-08-08

**Authors:** Yuki Ikeno, Yoshishige Takayama, Takashi Matsueda, Maiko Miyoshi, Tatsuo Motoki, Atsushi Kurushima, Takashi Otani, Yoshiaki Fukumura

**Affiliations:** 1 Department of Cardiovascular Surgery, Japanese Red Cross Hospital Tokushima, Komatsushima, Tokushima, Japan; 2 Division of Simcenter Customer Support, Department of CCM, Siemens.K.K, Tokyo, Japan

**Keywords:** Computational fluid dynamics, Total arch replacement, Aortic dissection

## Abstract

**Background:**

The indication of additional aortic arch surgery for residual aortic dissection remains controversial as some patients experience aneurysm rupture at a smaller diameter of 55 mm.

**Method:**

An 84-year-old woman, who underwent total arch replacement for chronic dissection, developed rupture of a residual aneurysm of with a diameter of 48 mm. Computational fluid dynamics simulated pressure and wall shear stress comparing pre- and post-total arch replacement.

**Results:**

After total arch replacement, false lumen pressure in the distal aortic arch increased (pre, 138.5 mmHg; post, 148.2 mmHg). Wall shear stress also increased in the distal aortic arch (pre, 10.5 Pa; post, 16.9 Pa).

**Conclusion:**

Computational fluid dynamics could retrospectively predict a significant postoperative increase in false lumen pressure and wall shear stress of chronic dissections after total arch replacement, potentially leading to aneurysm rupture.

**Supplementary Information:**

The online version contains supplementary material available at 10.1186/s44215-023-00091-w.

## Introduction

With improved surgical outcomes for acute DeBakey type I aortic dissection (AIAD), prevention of late aortic rupture is essential after initial aortic surgery, as 12–24% of hospital survivors die of distal aortic rupture [1]. Our threshold for additional aortic surgery is a descending aortic diameter of > 55 mm in chronic dissection patients [2]. However, the indication remains controversial as some patients experience aneurysm rupture at a smaller diameter, particularly in small elderly patients [2].

We report a case of chronic dissection rupture with a diameter of 48 mm after total arch replacement (TAR) for a dilated aortic aneurysm after the initial hemiarch for AIAD. Computational fluid dynamics (CFD) revealed the potential cause of the rupture, which provided helpful insight into preventing aneurysm rupture.

## Case

An 84-year-old woman underwent TAR via re-sternotomy due to a residual arch dissection of 55 mm, 11 years after the initial hemiarch for AIAD (Supplementary Figure S1). Preoperative computed tomography (CT) revealed that there was a large entry tear at the distal anastomosis of hemiarch. The re-entry tear occurred at the infrarenal abdominal aorta. TAR was performed by selective antegrade cerebral perfusion under moderate hypothermia. Single-barrel anastomosis of the true lumen (TL) at Zone 3 was performed for a distal anastomosis using a 24-mm four-branched graft (J-Graft, Japan Lifeline, Tokyo) and a Teflon felt strip. Elephant trunk (ET) was not inserted due to relatively small true lumen. The postoperative course was uneventful; however, postoperative CT revealed the detachment of the sutured flap of the distal anastomosis, which resulted in a double-barrel fashion (Supplementary Figure S2). Since the diameter of the distal aortic arch was 48 mm, the patient was discharged. Staged TEVAR was planned after evaluating diameter change in outpatient clinic.

The patient developed sudden chest pain and hypotension 6 weeks after TAR, and CT imaging showed a ruptured distal aortic arch with a hemothorax. Therefore, emergent total descending stent-grafting was performed (Gore C-TAG, 28 mm × 10 cm, 28 mm × 15 cm, 28 mm × 20 cm ® W. L. Gore & Associates, Inc., Newark, DE, USA), and postoperative CT revealed hemostasis. She was discharged to a nursing home 1 month after stent grafting without complications.

### CFD simulation

**Fig. 1:**
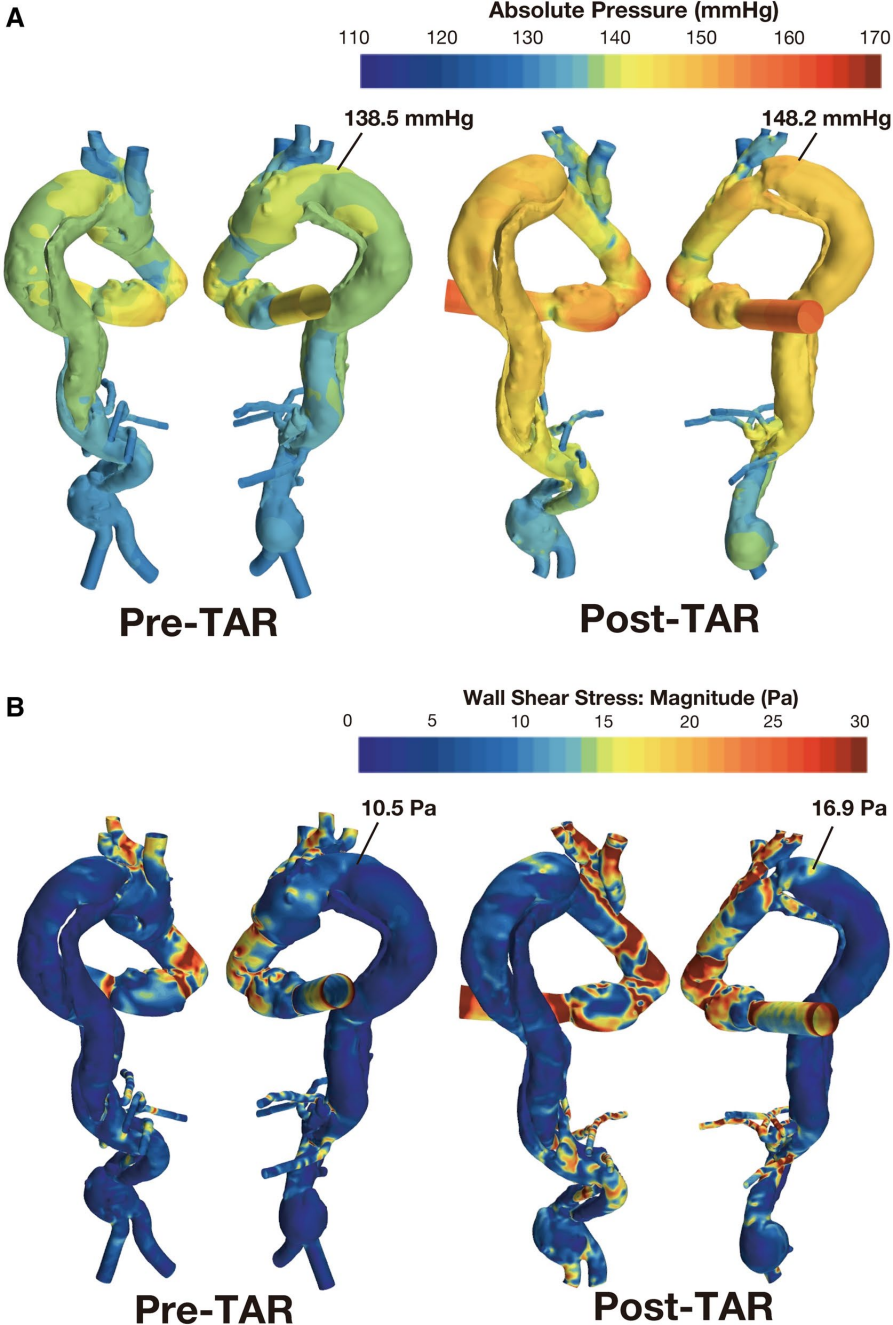
Computational fluid dynamics for aortic wall pressure and wall shear stress in systole comparing pre-TAR and post-TAR. **A** False lumen pressure increased at the distal aortic arch after total arch replacement (pre-TAR 138.5 mmHg; post-TAR, 148.2 mmHg). **B** False lumen wall shear stress increased at the distal aortic arch after total arch replacement (pre-TAR, 10.5 Pa; post-TAR, 16.9 Pa). Abbreviations: pre-TAR, pre-total arch replacement; post-TAR, post-total arch replacement

**Fig. 2:**
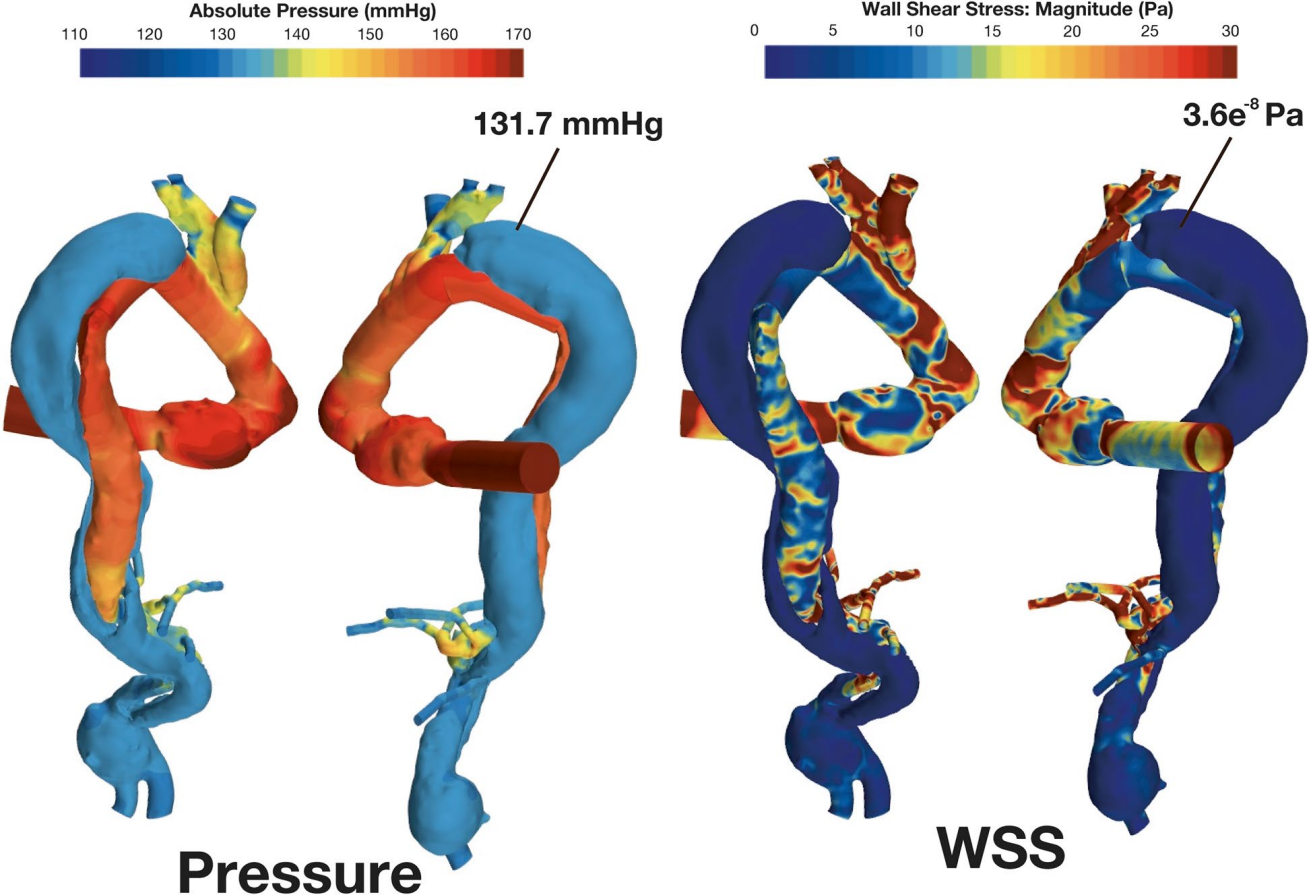
Computational fluid dynamics for aortic wall pressure and WSS in systole simulating elephant trunk insertion. False lumen pressure and WSS decreased if the elephant trunk was inserted in the true lumen (false lumen pressure, 131.7 mmHg; WSS 0 Pa). Abbreviations: WSS, wall shear stress

Patient-specific CFD based on her CT images retrospectively simulated aortic wall pressure and wall shear stress (WSS) in systole (Fig. 1A, B) (Supplementary Figure S3). A steady-state analysis was performed to simulate the maximum flow condition in a beating flow. The Stereolithography (STL) files converted from the Digital Imaging and Communications in Medicine (DAICOM) data of the CT were used to create the mesh used in the analysis. Detailed conditions and concepts of CFD are described in Supplemental Figure S4. After TAR, FL pressure in the distal aortic arch increased (pre-TAR, 138.5 mmHg; post-TAR, 148.2 mmHg). Moreover, after TAR, WSS increased in the distal aortic arch (pre-TAR, 10.5 Pa; post-TAR, 16.9 Pa). The CFD simulating ET for aortic wall pressure and WSS in systole are shown in Fig. 2. When the ET was inserted into the TL, FL pressure, and WSS decreased in the distal aortic arch after performing TAR (FL pressure, 131.7 mmHg; WSS, 3.6e^−8^ Pa) (Supplementary Figure S4).

## Discussion

Patient-specific geometrical vascular models for CFD—including CT and magnetic resonance imaging—are emerging technologies, particularly for ischemic heart disease and cerebral artery aneurysms [3]. However, predicting future adverse events remains challenging in the field of aortic disease [4].

The present study highlights the following findings: (1) FL pressure and WSS significantly increased after TAR with double-barreled distal anastomosis, and postoperative changes in flow direction might lead to aneurysm rupture, even in a small aneurysms; and (2) FL pressure and WSS could decrease when the ET was inserted. These CFD findings of the patient might be able to facilitate earlier intervention before rupture. Indeed, fluid dynamics should be considered in addition to morphological features such as diameter, area, and FL status. This case report does not provide a cutoff value of FL pressure and WSS. However, we believe that compared with preoperative parameters, postoperative increased FL pressure and WSS can be a risk factor of aneurysm rupture. Since the CFD analyses were performed by using routine CT imaging, the high availability of CFD is an advantage for clinical application.

Although our simulations could predict clinical outcomes, there is a lack of analysis of peripheral arterial resistance and small re-entries. In addition, ET simulation was performed using postoperative TL configuration (approximately 22 mm graft). The gap between simulation and real world should be consider. Therefore, the reliability of the results is limited, and clinicians should consider other information that influences the disease.

## Conclusions

CFD could retrospectively predict a significant postoperative increase in false lumen pressure and WSS of chronic dissections after TAR, potentially leading to aneurysm rupture, virtual simulation of elephant trunk insertion might be able to reduce false lumen pressure and WSS, and CFD may provide helpful information for decision-making regarding the optimal timing and procedure.

## Supplementary Information

**Additional file 1:** **Supplementary Figure S1.** Pre-total arch replacement computed tomography.

**Additional file 2: Supplementary Figure S2.** Post-total arch replacement computed tomography.

**Additional file 3: Supplementary Figure S3.** Detailed conditions of computational fluid dynamics.

**Additional file 4: Supplementary Figure S4.** Streamline.

## Data Availability

The datasets used and/or analyzed during the current study are available from the corresponding author on reasonable request.

## Abbreviations

AIAD: DeBakey type I aortic dissection
TAR: Total arch replacement
CFD: Computational fluid dynamics
CT: Computed tomography
TL: True lumen
FL: False lumen
WSS: Wall shear stress
