# Supplementary figures and images for: Computational fluid dynamics-based prediction of aortic aneurysm rupture in a patient with chronic aortic dissection
Source: Gen Thorac Cardiovasc Surg Cases. 2023 Aug 8;2:80. doi: 10.1186/s44215-023-00091-w (PMC11533624; doi:10.1186/s44215-023-00091-w)

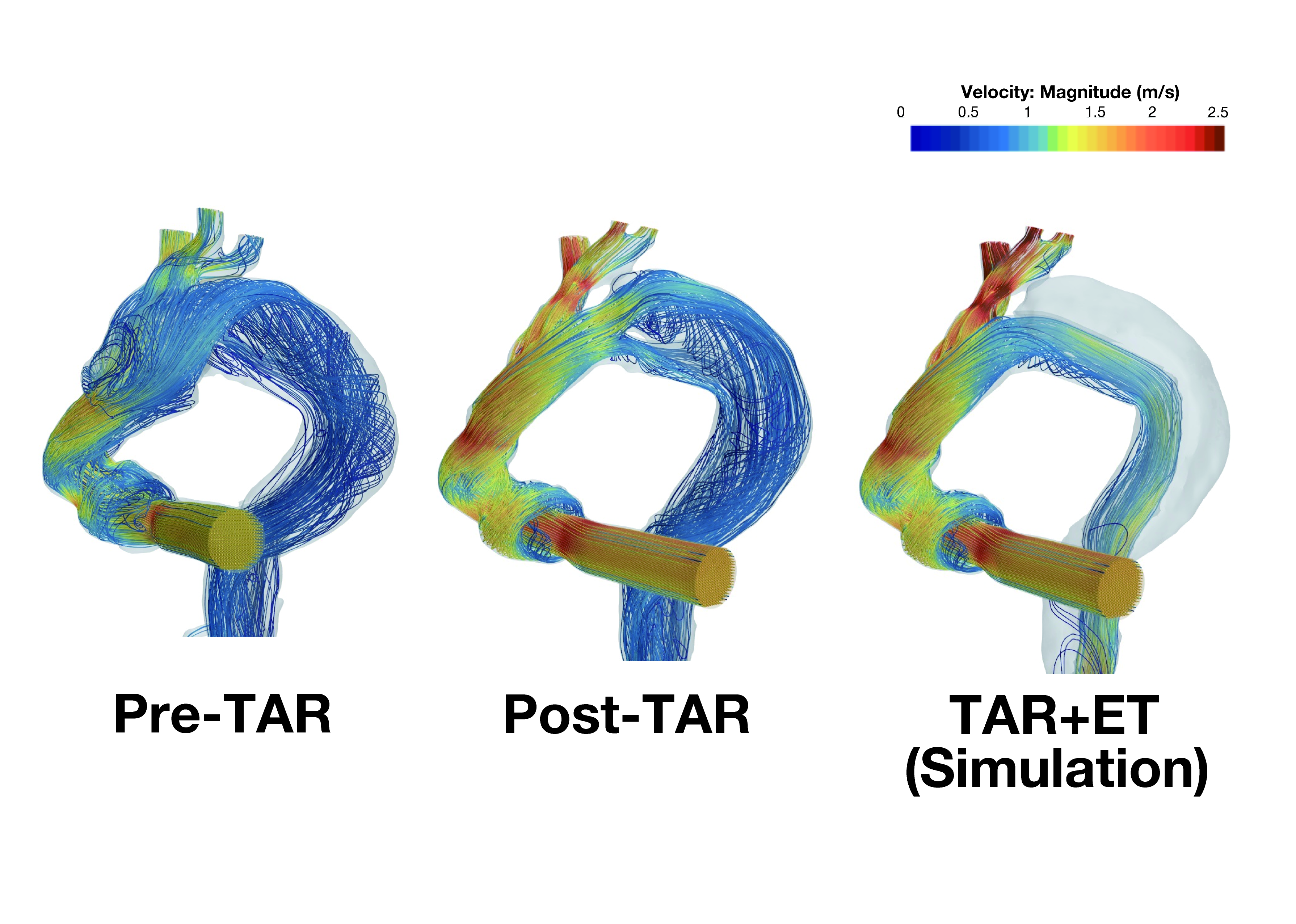

Supplement: Supplementary file 3 — Additional file 3: Supplementary Figure S3. Detailed conditions of computational fluid dynamics. [file 44215_2023_91_MOESM3_ESM.tif]

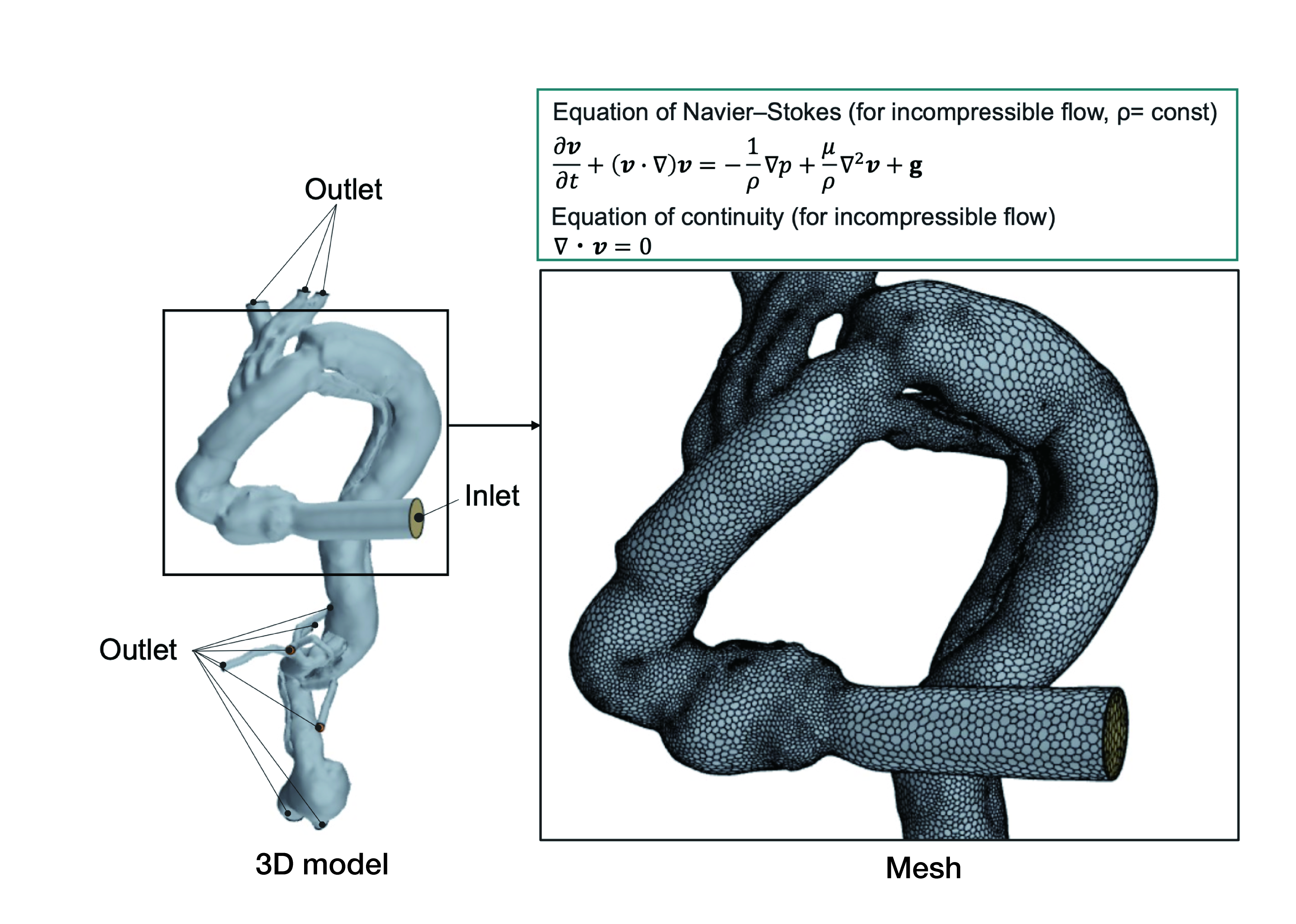

Supplement: Supplementary file 4 — Additional file 4: Supplementary Figure S4. Streamline. [file 44215_2023_91_MOESM4_ESM.tif]
